# Left ventricular hypertrophy among black hypertensive patients: focusing on the efficacy of angiotensin converting enzyme inhibitors

**DOI:** 10.1186/1756-0500-7-45

**Published:** 2014-01-20

**Authors:** Gari Negeri Jaleta, Esayas Kebede Gudina, Wondim Getinet

**Affiliations:** 1 Department of internal medicine, Jimma University, Jimma, Ethiopia; 2 Department of radiology, Jimma University, Jimma, Ethiopia

**Keywords:** Hypertension, Left ventricular hypertrophy, Cardiovascular disease, Angiotensin converting enzyme inhibitors, Ethiopia, Africa

## Abstract

**Background:**

Left ventricular hypertrophy (LVH) is an independent cardiovascular risk factor in patients with essential hypertension. The main objective of this study was to assess the echocardiographic prevalence of left ventricular hypertrophy in patients with hypertension, its risk factors and effect of antihypertensive drugs on its prevalence.

**Methods:**

A hospital based cross sectional study was conducted on 200 hypertensive patients on treatment in southwest Ethiopia. A pretested structured questionnaire was used to collect data from participants and their clinical records. Blood pressure and anthropometric measurements were taken according to recommended standards. Left ventricular mass was measured by transthoracic echocardiography. Associations between categorical variables were assessed using chi-square test and odds ratio with 95% confidence interval. Logistic regression model was done to identify risks factors of LVH. P values of < 0.05 were considered as statistically significant.

**Results:**

The mean age, systolic blood pressure, diastolic blood pressure and body mass index were 55.7 ± 11.3 years, 139.2 ± 7.7 mmHg, 89.2 ± 5.7 mmHg and 24.2 ± 3.4 Kg/m^2^ respectively. The overall prevalence of LVH among these study subjects was 52%. Age ≥50 years (OR: 3.49, 95% CI 1.33-9.14, P = 0.011), female gender (OR: 7.69, 95% CI 3.23-20.0, P < 0.001), systolic blood pressure ≥140 mmHg (OR: 2.85, 95% CI 1.27-6.41, P = 0.011), and duration of hypertension (OR: 3.59, 95% CI 1.47-8.76, P = 0.005) were independent predictors of left ventricular hypertrophy. Angiotensin converting enzyme (ACE) inhibitors were the only antihypertensive drugs associated with lower risk of left ventricular hypertrophy (OR: 0.08, 95% CI 0.03-0.19, p < 0.001).

**Conclusions:**

Left ventricular hypertrophy was found to be highly prevalent in hypertensive patients in Ethiopia. ACE inhibitors were the only antihypertensive drugs associated with reduced risk of LVH. We thus recommend strategies to early detect and treat hypertension and to timely screen for LVH among patients with hypertension. Multicenter prospective studies in Africa settings would be ideal to identify the best antihypertensive agents in black Africans.

## Background

Cardiovascular disease (CVD) is the leading cause of death globally resulting in about 30% of deaths annually. About half of CVD related deaths are attributable to hypertension, a disease strongly associated with overall cardiovascular risk [1].

Long standing hypertension results in left ventricular hypertrophy (LVH), a preclinical cardiac damage that in the long run may lead to potentially life-threatening complications [2,3]. LVH is strongly associated with major cardiovascular events [4-11]. Its echocardiographic prevalence varies from around 20% to over 70% based on the criteria used and population studied [4,7,12-14].

Cardiac hypertrophy is a compensatory response to cardiac insult of any cause [13]. It is the phenotypic consequence of interactions between genetic and non-genetic factors that involve multiple etiologies and complex mechanisms. Genes encoding proteins involved in the structure of the left ventricle (LV) and genes encoding cell signal transduction, hormones, growth factors, calcium homeostasis, and blood pressure are likely candidates for the development of LVH [15]. However, identification of specific genetic and molecular mechanisms for LVH in hypertensive patients is challenging [15-17]. For instance, CaMK4 gene deletion in mice was found to be linked with occurrence of hypertension, resultant LVH and reduced survival. However, treatment with furosemide in such mice prevented the development of both hypertension and LVH suggesting that LVH is a result of increased BP rather than being genetically determined [16].

Despite these challenges, the pathophysiologic mechanisms of LVH appear to be well established now. LVH is a myocardial remodeling [13] that ensues through activation of different signaling pathways [15] which involve mechanical [18] and neurohormonal activation by agents such as catecholamines and vasoactive peptides (angiotensin II or the endothelins) [19,20]. The benefits of beta-blockers and renin-angiotensin aldosterone system inhibitors in the treatment of CVDs are thus derived from their inhibition of these neurohormonal mechanisms [13]. Their importance in heart failure in particular has recently been elaborated [21].

Secondary messengers that regulate nuclear transcription factors activity (NF-κB, CREB, NFAT, and GATA-4) are activated in response to the neurohormonal activation. The nuclear transcription factors modify the expression of hypertrophic genes [22,23]. NF-κB is the most prominent of these transcription factors that modulate cellular response in myocardial remodeling [24]. This was shown by inhibition of NF-κB transcription activity by intracardiac injection of AdGRK5-NT that reduced LVH. Treatment with captopril (angiotensin converting enzyme inhibitor) also significantly reduces the heart size and inhibits NF-κB activity [25].

Though different mechanisms interplay in the development of LVH, there is a consistent and independent interaction between hypertension and left ventricular mass (LVM) [3]. However, the relationship between degree of hypertension and LVM is not linear. LVH may occur in patients with borderline hypertension. It is a major risk factor for coronary artery disease all-cause mortality even in the absence of symptoms and other cardiovascular risk factors [26]. The effect is even more prominent in black patients [27] where it was found to be associated with greater relative and attributable risk than the traditional risk factors for coronary disease [28].

Apart from hypertension itself, different factors are believed to play a role in the development of LVH. Obesity, dyslipidemia, diabetes mellitus, smoking, old age and excessive alcohol intake are said to be positively correlated with the prevalence of LVH in hypertensive patients [12,29-32].

Studies regarding LVH and its sequel are scarce in Black Africans. Most prevalence data for hypertension and LVH as well as treatment recommendations are extrapolated from the findings in African Americans. The main aim of the current study is thus to assess the prevalence of left ventricular hypertrophy in Ethiopian hypertensive patients and role of ACE inhibitors in preventing LVH in patients with hypertension.

## Methods

### Settings

This is a cross-sectional study conducted at the Jimma University Specialized Hospital (JUSH) between May and August 2012. JUSH is a referral hospital for around 15 million people in southwest Ethiopia. It is one of the few teaching hospitals in the country. It is located in Jimma town, 356 km south-west of the capital Addis Ababa. At the time of the study, there were around 1100 patients on active follow-up for hypertension at hypertension clinic of the hospital. The clinic runs once a week every Wednesday. The service is rendered by internists, medical residents and medical interns.

### Selection of participants

Hypertensive patients coming for follow-up to the hospital were consecutively recruited based on the selection criteria and their willingness to participate in the study. Over a period of four months, complete data from the patient interview, physical examination, chart review and echocardiographic assessment was done in 200 hypertensive patients.

### Inclusion criteria

Any adult hypertensive patient over 18 years of age coming to the clinic for follow up was enrolled for the study based on their willingness and consent.

### Exclusion criteria

All hypertensive patients with heart failure were excluded from the study.

### Data collection process and instrument

The data were collected using a pre-tested structured questionnaire which was prepared specifically for this study. Patient interviews, BP and anthropometric measurements were done by nurses working in the clinic after one day of training. Chart review was done by medical residents. The questionnaire consisted of socio-demographic profiles; physical measurements (blood pressure, weight, height) and clinical records for co-morbidities, duration of hypertension and the types of antihypertensive medications used. Blood pressure was measured with a sphygmomanometer. An average of three blood pressure measurements was taken to assess blood pressure control (the current and the last two visits). Height and weight were measured using a standard weighing scale and stadiometer respectively. These parameters were measured by patient standing straight without shoes and wearing only light clothes.

Transthoracic echocardiography was performed to measure parameters used to estimate left ventricular mass (LVM). M-mode tracing was done at the papillary muscle level of the left ventricle to measure posterior wall thickness in diastole (PWTd), interventricular septal wall thickness in diastole (IVSTd) and left ventricular internal diameter in diastole (LVIDd). Absolute LVM was calculated by the Devereux formula given as 0.8 * 1.04 ((PWTd cm + IVSTd cm + LVIDd cm)^*3*^*– LVIDd*^*3*^*cm )) + 0.6 grams*[33]. The LVM was indexed to body surface area (BSA). LVH was defined as LVM/BSA of > 116 g/m^2^ for men and > 96 g/m^2^ for women [33].

### Data analysis

Data were analyzed using SPSS version 20. The findings were expressed as mean ± standard deviation and/or percentages. Categorical variables were compared using Chi-square test and odds ratio with 95% confidence interval. A P-value of less than 0.05 was considered statistically significant. The logistic regression model was done to identify predictors/risks of left ventricular hypertrophy.

### Ethical considerations

Ethical clearance was obtained from Jimma university ethical review board. Informed written consent was obtained from every participant. The information collected from participants will remain confidential indefinitely. Patients with life threatening conditions were exempted from the study and were linked for appropriate care.

## Results

### Background characteristics

A total of 200 hypertensive patients were included in the study of which 57% were women. The mean age of the participants was 55.7 ± 11.1 years with a range of 22 to78 years. Hundred forty-nine (74.5%) of them were older than 50 years. About 70% of them had hypertension for 10 years or more. The mean systolic blood pressure (SBP) and diastolic blood pressure (DBP) were 139.2 ± 7.7 mmHg and 89.2 ± 5.7 mmHg respectively (Table 1).

**Table 1:** Background Characteristics of hypertensive patients in Ethiopia

**Characteristics**	
Age (years), Mean ± SD	55.7 ± 11.1
Age category, N (%)	
<50	51 (25.5)
≥50	149 (74.5)
Sex, N (%)	
Male	86 (43.0)
Female	114 (57.0)
Duration of hypertension, N (%)	
<10 yrs	60 (30.0)
≥10years	140 (70.0)
History of diabetes	
Yes	39 (19.5)
No	161 (80.5)
Blood pressure	
SBP (mmHg ), Mean ± SD	139.23 ± 7.79
DBP (mmHg), Mean ± SD	89.24 ± 5.75
BMI (Kg/m^2^), Mean ± SD	24.22 ± 3.47
BMI (Kg/m^2^), N (%)	
<25	130 (65.0)
25 – 29	56 (28.0)
≥30	14 (7.0)

SBP – systolic blood pressure, DBP – diastolic blood pressure.

BMI – Body mass index.

Thirty-nine (19.5%) of patients have been diagnosed with diabetes and were on glucose lowering agents (insulin or oral glucose lowering agent), 28 of them had type 2 diabetes.

None of the participants reported smoking or use of alcohol.

The mean Body Mass Index (BMI) was 24.2 ± 3.4 Kg/m^2^. Fifty-six (28%) of them were found to be overweight and 14 patients (7%) were classified as obese according to the standard classification of overweight as BMI ≥25 Kg/m^2^ and obesity as BMI ≥30 kg/m^2^ (Table 1).

### Treatment of hypertension

All of the participants were taking some types of antihypertensive agents at the time of the study. About 57% of them were taking a combination of two or more antihypertensive drugs. The most commonly used drug was hydrochlorothiazide used in 53% of the participants as the only agent or in combination with others. About 42% of them were taking angiotensin converting enzyme (ACE) inhibitors alone or in combination with others. Beta-blocker (atenolol) alone or in combination with other agents was used by 38% of the participants. None of the patients were taking angiotensin receptor blockers (ARB) (Table 2).

**Table 2:** Types of anti-hypertensive drugs used by hypertensive patients in Ethiopia, August 2012

**Types of anti-hypertension drug**	**N**	**%**
Thiazide diuretic only	40	20.0
ACE inhibitor only	32	16.0
Beta blocker only	15	7.5
ACE inhibitor + Thiazide diuretic	18	9.0
Beta blocker + Thiazide diuretic	23	11.5
Calcium channel blocker + Thiazide diuretic	7	3.5
ACE inhibitor + Beta blocker	11	5.5
ACE inhibitor + Calcium channel blocker	4	2.0
Calcium channel blocker + Beta blocker	3	1.5
Three drugs (different combinations)	47	23.5

### Echocardiographic findings and the Prevalence of left ventricular hypertrophy

The mean posterior wall thickness in diastole (PWTd), interventricular septal wall thickness in diastole (IVSTd) and left ventricular internal diameter in diastole (LVIDd) were 1.28 ± 0.25, 1.38 ± 0.31and 3.93 ± 0.4 centimeters respectively. The mean calculated left ventricular mass from the three parameters according to the Devereux formula was 193.4 ± 60.6 grams. When indexed to BSA, it was 107.3 ± 33.6 g/m^2^.

The overall prevalence of left ventricular hypertrophy based on criteria of LMV/BSA >116 g/m^2^ for men and >96 g/m^2^ for women was 52%. There was a significant difference in its prevalence between genders, 68.4% among women and 30.2% among men (p < 0.001) (Table 3).

**Table 3:** Bivariate analysis of risk factors for Left ventricular Hypertrophy among hypertensive patients in Ethiopia

**Predictor variables**		**LVH ( N = 200 )**		**P-value**
		**Yes, N (%)**	**No, N (%)**	
Sex	Female	78(68.4)	36(31.6)	<0.001
	Male	26(30.2)	60(69.8)	
Age	≥50 years	92(61.7)	57(38.3)	<0.001
	<50 years	12(23.5 )	39(76.5)	
BMI	≥25 Kg/m^2^	40(57.1)	30(42.9)	0.172
	<25 Kg/m^2^	64(49.2)	66(50.8)	
SBP	≥140 mmHg	65(63.7)	37(36.3)	<0.001
	<140 mmHg	39(39.8)	59(60.2)	
DBP	≥90 mmHg	56(56.0)	44(44.0)	0.258
	<90 mmHg	48(48.0)	52(52.0)	
History of DM	Yes	20(51.3)	19(48.7)	0.920
	No	84(52.2)	77(47.8)	
Type of DM	Type 2	15(53.6)	13(46.4)	0.897
	Type 1	5(45.5)	6(54.5)	
ACE inhibitor	No	86(74.1)	30(25.9)	<0.001
	Yes	18(21.4)	66(78.6)	
Beta Blocker	Yes	43(57.3)	32(42.7)	0.242
	No	61(48.8)	64(51.2)	
Duration of hypertension	≥10 years	88(62.9)	52(37.1)	<0.001
	<10 years	16(26.7)	44(73.3)	
Thiazide diuretic	Yes	54(50.9)	52(49.1)	0.751
	No	50(53.2)	44(46.8)	
Calcium channel blocker	Yes	9(47.4)	10(52.6)	0.671
	No	95(52.5)	86(47.5)	

DM – diabetes mellitus, ACE – angiotensin converting enzyme.

### Risk factors for the development of left ventricular hypertrophy

#### Age

Of the 104 patients with left ventricular hypertrophy, 92 (88.4%) were 50 years or older. A bivariate analysis showed that age ≥50 years was associated with the development of LVH (crude OR: 5.24 95% CI 2.53- 10.84, P < 0.001) (Table 3). When age was adjusted for other variables in logistic regression, it remained an independent risk factor for the development of LVH (adjusted OR: 3.49, 95% CI 1.33-9.14, P = 0.011) (Table 4).

**Table 4:** Independent risk factors for Left ventricular Hypertrophy among hypertensive in Ethiopia

**Risk factors**	**Adjusted OR**	**95% CI for OR**		**P-value**
Age	3.49	1.33	9.14	0.011
Sex (female)	7.69	3.23	20.0	<0.001
Systolic BP	2.85	1.27	6.41	0.011
ACE inhibitor	0.08	0.03	0.19	<0.001
Duration of hypertension	3.59	1.47	8.76	0.005

#### Gender

The prevalence of LVH in women was found to be 68.4% versus 30.2% in men. Bivariate analysis showed that females were 5 times more likely to develop LVH as compared to their male counterparts (crude OR: 5.0, 95% CI 2.72- 9.16, P < 0.001) (Table 3). Controlling for other potential risk factors on logistic regression, this effect remains significant (adjusted OR = 7.69(3.23-20.0), P < 0.001) (Table 4).

#### BMI

Overall, 70 (35%) of the participants had BMI ≥25 Kg/m^2^. However, there was no significant difference in the proportion of LVH between individuals with normal or high BMI (P = 0.172).

#### Diabetes mellitus

Diabetes was not found to be a significant predictor of LVH in this study. All of the 28 type 2 patients were taking oral glucose lowering agents as all type 1 patients were on insulin. However, neither the type of diabetes nor the treatment was associated with LVH.

#### Degree of blood pressure control

At the time of this study, about 60% of the participants had high blood pressure defined as SBP ≥ 140 mmHg and/or DBP ≥ 90 mmHg. Significant difference in prevalence of LVH was detected only when dichotomizing the high BP into SBP or DBP. Elevated systolic blood pressure was found to be an independent predictor of LVH in patients with hypertension, (adjusted OR: 2.85, 95% CI 1.27–6.41, p = 0.011). However, a significant interaction between DBP and LVH was not seen (p = 0.258) (Tables 3 and 4).

#### Duration of hypertension

About 70% of the participants reported that they had hypertension for 10 years or more. The prevalence of LVH in this group was 62.9% versus 26.7% in those who were less than 10 years since diagnoses. Adjusting for all potential risk factors, patients with longstanding hypertension (≥10 years) were 3.59 times more likely to develop LVH as compared to those with less than 10 years of hypertension (adjusted OR: 3.59, 95% CI 1.47- 8.76, P = 0.005) (Table 4).

#### Types of antihypertensive agent used and the risk of LVH

Comparison of LVM index was done among each antihypertensive agent used. Apparently lower LVM was seen in patients taking ACE inhibitors alone or in combination with other agents at the time of the study. The findings were comparable among other antihypertensive agents. Multivariate logistic regression also showed a decreased risk of LVH (adjusted OR: 0.08, 95% CI 0.03-0.19, p < 0.001) for those on ACE inhibitors (Table 4).

ACE inhibitors were used more widely in patients older than 50 years (66.4%) and in those with diabetes (82%). These agents were used less often (<50%) in other groups. The effect of ACE inhibitors on LVH was independent of these groupings. However, this effect vanished in those with high blood pressure (SBP ≥160).

When we grouped antihypertensive agents to those with anti-remodeling effect (ACE inhibitors and beta-blockers) and non-anti-remodeling drugs (other groups), a positive trend towards anti-remodeling agents was seen though not statistically significant (adjusted OR: 0.11, 95% CI 0.09-1.02, p < 0.001).

## Discussion

Left ventricular hypertrophy, a preclinical cardiac damage, was found to be highly prevalent in Ethiopian hypertensive patients. Age, female gender, longstanding and systolic hypertension were found to be independent predictors for its occurrences. Use of ACE inhibitors was associated with decreased risk of LVH. This cross-sectional study from a single health facility in Africa may give clues to the effect of ACE inhibitors in the treatment of hypertension and their effect on LVH in black patients.

LVH is an independent cardiovascular risk factor [3]. Its prevalence in hypertensive patients in most literatures worldwide varies from 20 to 70% based on the population studied and the criteria used [7,13,14,34]. The overall prevalence of left ventricular hypertrophy of 52% among hypertensive subjects in our study lies within this range.

Age was found to be an independent risk factor for LVH in most studies [7,8,35,36]. The finding in our study is also consistent with these findings.

Female gender was found to be an independent risk factor for LVH in hypertensive patients in our study with adjusted OR of 7.69. Different studies from around the world have shown that the gender effect on prevalence of LVH varies from population to population [35-39], whether ECG or echocardiography is used for its assessment [7] and criteria used [34]. A study in Pakistan, for instance, revealed that women were 11.35 times more likely to develop LVH than the male counterparts even after adjusting for other factors [37]. Similarly, studies in Nigeria [38], China [12] and the LIFE (losartan intervention for endpoint reduction in hypertension) study [39] showed a trend towards a higher prevalence in women. However, some studies from European countries have shown a higher prevalence in men [35,36]. Whether these differences are due to population studied or criteria used or cutoff points used need to be addressed in future studies.

The presences of diabetes [40] and high BMI [8,9] have shown significant correlations with LVH in most studies. However, in our study, neither history of diabetes nor the type of treatments for hyperglycemia showed any association with LVH. Our assessments for diabetes were based on only self-reported diagnosis without blood glucose test. Besides, the small sample size might not be enough to see this correlation. Absence of correlation between LVH and body habitus in our study can also be explained by the small sample size.

Uncontrolled systolic blood pressure ≥140 mmHg was associated with the development of left ventricular hypertrophy in this study. However, no correlation was found between diastolic blood pressure and the occurrence of LVH. These findings are consistent with results from other studies [41]. The reason for this difference could be due to the fact that diastolic hypertension is associated more with diastolic dysfunction than systolic dysfunction and LVH [41].

Duration of hypertension was found to be an independent predictor of left ventricular hypertrophy in our study which is consistent with most studies from around the world [42,43].

The fact that all of our study participants were nonsmokers and nonalcoholic was merely a chance occurrence. This can also be explained by the fact that the prevalence of smoking in Ethiopia is low, 3.3% in the general population and 0.5% for women [44] who made for the larger part of our study participants. In addition, as most of our participants had a diagnosis of hypertension for over 10 years and all have been on treatment for their hypertension, abstinence from smoking for medical reason may also explain our findings.

As LVH is a cardiac remodeling problem [5,13], we tried to evaluate the effect of anti-remodeling drugs (beta-blockers and ACE inhibitors) on the risk of LVH. It was found that patients using ACE inhibitors at the time of the study had a lower LVM index and decreased risk of LVH as compared to other agents. The effect of beta-blockers was however less pronounced. Evidences have shown that anti-remodeling agents like beta-blockers, ACE inhibitors and ARB have protective effect against development of LVH. Besides, they can even reverse an established LVH and improve left ventricular function [45-49]. We understand that such cross-sectional study like ours may not be enough to see the full effect of these drugs on regression of LVH.

In addition to this, recent studies have also casted doubts on the benefits of these drugs among black patients [50]. Such evidences showed that ACE inhibitors, ARB and beta-blockers are less effective than calcium channel blockers and diuretics in black hypertensive patients with or without LVH due low plasma renin level in blacks [50,51]. However, most of these studies were extrapolated from findings in African-Americans. Multicenter studies in indigenous Africans would be ideal to identify best antihypertensive drug options in such setting where the dietary habits and physique are so diverse (for instance, West Africans versus East Africans).

Another explanation for the lack of effect of beta-blockers in our study may be due to the fact that most (56%) of the patients on these agents had high SBP (≥160 mmHg). In these patients, beta-blockers were used as additional antihypertensive agents after blood pressure control became difficult with original one or two types of antihypertensive drugs. It should also be reminded here that the benefit of beta-blockers on regression of LVH and uncomplicated hypertension is becoming questionable [52]. In addition to this, carvedilol is the beta-blocker of choice in patients with hypertensive LVH [53]. The beta-blocker used in our study was however atenolol.

The effect of ACE inhibitors on LVH also vanished in those with SBP ≥ 160 mmHg. This is contrary to the fact that ACE inhibitors induce left ventricular hypertrophy regression, independent of changes in blood pressure [54]. Cross-sectional study like ours may not be enough to see these effects.

Even though this study has come up with such important findings, there are important limitations worth mentioning here. First of all, the relatively small sample size might have contributed to the inability to detect the effect of certain risk factors for LVH. For example none of the participants reported a history of smoking or alcohol use. On the other hand, other potential risk factors for LVH like dyslipidemia, anemia and renal dysfunction were not assessed making an assessment of risk factors incomplete. Finally, as all of the participants were those on pharmacological treatment for hypertension, it may not be generalizable for all patients with hypertension in the country.

## Conclusion

Left ventricular hypertrophy was found to be highly prevalent in Ethiopian hypertensive patients where BP control is suboptimal. Older age, female gender, longstanding and systolic hypertension were independent predictors of LVH. ACE inhibitor use on the other hand has been associated with lower risk of this complication. Taking these results and observations into consideration, we thus recommend early screening of hypertensive patients for LVH with either echocardiography or electrocardiography based on availability. Besides, we urge health care providers and policymakers to improve standards of care for hypertensive patients by optimizing BP control and working on those modifiable risk factors of LVH.

Last but not least, we recommend a large scale multicenter study to substantiate the findings in this research.

## Competing interests

The authors declare that there is no conflict of interest associated with this research work.

## Authors’ contributions

GNJ designed the study, developed the instruments, supervised data collection, analyzed the data and wrote the manuscript. EKG participated in study design, instrument development, data analysis and writing of manuscript. WG participated in study design and instrument development, took part in data collection and editing manuscript. All authors read and approved the final manuscript.
